# Contracting-out primary health care services in Tanzania towards UHC: how policy processes and context influence policy design and implementation

**DOI:** 10.1186/s12939-018-0835-8

**Published:** 2018-10-05

**Authors:** Stephen Maluka, Dereck Chitama, Esther Dungumaro, Crecensia Masawe, Krishna Rao, Zubin Shroff

**Affiliations:** 1 Institute of Development Studies, University of Dar es Salaam, P.O.BOX 35169, Dar es Salaam, Tanzania; 2 Muhimbili University of Health and Allied Sciences, Dar es Salaam, Tanzania; 3 Dar es Salaam University College of Education, Dar es Salaam, Tanzania; 4 Bloomberg School of Public Health, Johns Hopkins University, Baltimore, USA; 5 Alliance for Health Policy and Systems Research, World Health Organization, Geneva, Switzerland

**Keywords:** Contracting-out, Non-state providers, Primary health care, Tanzania

## Abstract

**Background:**

Governments increasingly recognize the need to engage non-state providers (NSPs) in health systems in order to move successfully towards Universal Health Coverage (UHC). One common approach to engaging NSPs is to contract-out the delivery of primary health care services. Research on contracting arrangements has typically focused on their impact on health service delivery; less is known about the actual processes underlying the development and implementation of interventions and the contextual factors that influence these. This paper reports on the design and implementation of service agreements (SAs) between local governments and NSPs for the provision of primary health care services in Tanzania. It examines the actors, policy process, context and policy content that influenced how the SAs were designed and implemented.

**Methods:**

We used qualitative analytical methods to study the Tanzanian experience with contracting- out. Data were drawn from document reviews and in-depth interviews with 39 key informants, including six interviews at the national and regional levels and 33 interviews at the district level. All interviews were audiotaped, transcribed and translated into English. Data were managed in NVivo (version 10.0) and analyzed thematically.

**Results:**

The institutional frameworks shaping the engagement of the government with NSPs are rooted in Tanzania’s long history of public-private partnerships in the health sector. Demand for contractual arrangements emerged from both the government and the faith-based organizations that manage NSP facilities. Development partners provided significant technical and financial support, signaling their approval of the approach. Although districts gained the mandate and power to make contractual agreements with NSPs, financing the contracts remained largely dependent on donor funds via central government budget support. Delays in reimbursements, limited financial and technical capacity of local government authorities and lack of trust between the government and private partners affected the implementation of the contractual arrangements.

**Conclusions:**

Tanzania’s central government needs to further develop the technical and financial capacity necessary to better support districts in establishing and financing contractual agreements with NSPs for primary health care services. Furthermore, forums for continuous dialogue between the government and contracted NSPs should be fostered in order to clarify the expectations of all parties and resolve any misunderstandings.

## Background

Public health facilities in many low-and-middle-income countries (LMICs) often have limited human resources and provide inadequate access to health care for the population. These limitations are associated with inadequate improvements in health outcomes in recent decades. Governments seeking to move towards Universal Health Coverage (UHC) are increasingly recognizing that they need to engage non-state providers (NSPs) to address gaps in their health systems. NSPs include all health care providers outside government health facilities [1], including private-for-profit providers, private-not-for-profit providers, and informal providers such as traditional healers [2].

One common approach to engaging NSPs has been contracting with them to deliver primary health care services to a specified population on behalf of the government [3]. Typically, a formal contract is established between the government and one or more NSPs that stipulates the responsibilities of all parties involved in the contract, the type(s) of health care services to be provided, how the contract will be financed, and accountability and performance monitoring mechanisms.

In LMICs, the private for-profit and non-profit sectors represent important and often well-resourced providers of health care services. Governments are motivated to contract with these NSPs both to utilize all available resources to increase coverage of health services to the population and to improve the effectiveness and efficiency of services through fostering competition [2]. Contracting-out has also been encouraged by a range of external factors, including the need to quickly scale up vertical health programs, concerns about the quality of available health care services and the lack of adequate health care personnel in the public sector [2, 3].

The implementation of contracting-out may, however, be hampered in many LMICs by several factors, including high administrative costs and lack of sufficient providers for meaningful competition in rural areas. As elsewhere, existing vested interests among the parties involved in bidding on and awarding contracts may present other challenges to unbiased assessment and management of contracts [4]. Furthermore, contracting-out may result in further fragmentation of the health system, particularly in countries where monitoring is weak [3].

In Tanzania, NSPs of health services include faith-based organizations (FBOs), non-governmental organizations (NGOs), private for-profit providers and informal providers [5]. This paper focuses on faith-based providers, the most prominent group in terms of total infrastructure, number of staff, and geographic reach.

The private not-for-profit sector—of which the faith-based facilities make up the overwhelming majority—is the second largest provider of health services in the country [5]. The FBO sector owns 23.3% of health infrastructure, while the state owns 60%. However, 41.1% of hospitals are owned by FBOs while 40% are owned by the state, making faith-based NSPs the largest providers of hospital services in the country [5].

The Tanzanian government has a long history of providing subsidies to FBOs to serve areas without public health facilities. In 1992 the government formally negotiated agreements [6] to provide bed and staff grants to hospitals managed by FBOs. In districts without a government hospital, the government designated FBO hospitals to serve as District Designated Hospitals (DDHs). In these districts, the government provided operational support to hospitals owned by FBOs.

Since the introduction of the health sector reforms agenda in the 1990s, the concept of partnerships between government and NSPs for health services delivery has continued to gain importance. In 2005 the government revised the 1992 agreements. With this reform, district officials were empowered to contract with NSPs, with contracts to be signed at district level rather than by the Ministry of Health as had previously been the case. In 2007, reforms continued with the introduction of a new type of operational contract known as the Service Agreement (SA). This reform signaled the transition to a formal system backed up by solid legal frameworks and marked the end of basing contracts mainly on informal trust-based relationships [6].

Studies on contracting-out in LMICs have reported various, sometimes conflicting, experiences and evidence [7]. For example, in South Africa and Zimbabwe, contracted NSPs reportedly provided health care services of the same or higher quality at lower cost [7]. However, no significant performance differences were found between contracted and public providers in Ghana and Tanzania [7]. One review, which focused on the effectiveness of the contracting-out interventions in reaching poor and marginalized groups in low-and-middle-income countries, underscored a lack of robust evidence [8]. Another review, however, concluded that these interventions could be effective and should be scaled up with more robust evaluation [9]. Other reviews suggested that while contracting-out has improved access to health services, its effects on other performance areas—such as quality of services, efficiency and equity—remain inadequately understood [10, 11].

While at least some research has been done on impact, there is a paucity of knowledge on the actual processes underlying development and implementation of contracting-out interventions and the contextual factors that influence their performance. This paper reports on the design and implementation of SAs between local governments and NSPs for the provision of primary health care services in Tanzania. After examining the roles of actors, the policy process, the context and policy content that influenced how the SAs were designed and implemented, it discusses lessons regarding design and implementation of contracting out policy that may be useful learning for other countries. This study thus complements and explores in more depth the findings of a recent study on stakeholders’ perceptions regarding the Service Agreements (SA) [12].

## Methods

### Theoretical framework

This study uses the Walt and Gilson policy analysis triangle to frame our findings [13]. This enabled us to explicitly examine the roles of actors, policy processes and content, and contexts in explaining the design and implementation of contractual arrangements between local governments and NSPs. The policy analysis triangle recognizes that the health policy process involves four elements: the policy’s *content*; the *context* in which a policy is formulated and implemented; the *actors* involved in policy design and implementation; and the *processes* associated with policy design and implementation [13].

This analytical framework guided us in: mapping the processes involved in SA policy design and implementation; investigating how actors interacted and exercised financial, technical and political power [13] to influence the design and implementation of SAs; and assessing the nature and content of contracts, including the types of services covered, target population, financing, and accountability and performance monitoring mechanisms.

### Study setting

**Fig. 1:**
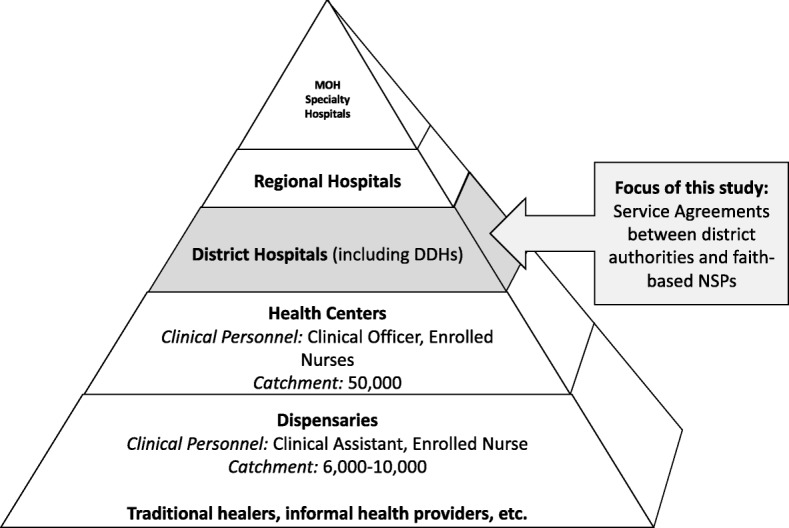
Structure of the health care system in Tanzania

Tanzania is a low-income country in sub-Saharan Africa with a population of 55.5 million. As in many other countries, the public health system is organized in the form of a pyramid (Fig. 1). Various forms of primary health care facilities make up the pyramid’s bottom. Dispensaries represent the lowest level of health care delivery in the country; they are supposed to be run by a clinical assistant and an enrolled nurse who offer basic outpatient curative care to a catchment of between 6000 and 10,000 people. Health centers serve populations of about 50,000 people; these are staffed by clinical officers supported by enrolled nurses. Further up the pyramid, district hospitals offer inpatient services and outpatient services not available at dispensaries or health centers. Most districts in Tanzania have a government-run district hospital. However, in districts without a public hospital, hospitals run by NSPs are designated district hospitals (DDH) and receive government subsidies—the bulk of NSP designated district hospitals are FBOs. Multiple districts are grouped into regions, each of which has a regional hospital. Finally, at the top of the pyramid are specialized hospitals owned by the Ministry of Health. This study focuses on SAs signed between district authorities and FBO hospitals [14].

Health sector funding comes from two main sources: central support financed by the government of Tanzania’s general tax revenue; and development partners’ (DPs) support. DPs provide pooled funding both through general budget support (GBS) and the Health Basket Fund (HBF), a form of sector budget support [15]. As indicated in Table 1, Tanzania depends on a significant level of DP support to finance health care, while the share of funding from domestic taxes remains low.

**Table 1:** Total Health Expenditures by Source (percent)

	FY2002/03	FY2005/06	FY2009/10	FY2011/12
Households	42%	25%	32%	27%
DPs	27%	44%	40%	47%
Ministry of Finance	25%	28%	26%	21%
Other	6%	3%	2%	5%
TOTAL	100%	100%	100%	100%

Source: National Health Accounts (2014)

Governance of the health system occurs at multiple levels. The Ministry of Health, Community Development, Gender, Elderly and Children (abbreviated either as MoHCDGEC or Ministry of Health) is mandated to provide overall stewardship of the health sector. This Ministry is responsible for policy development, strategic planning, resource mobilization, and monitoring and evaluation. Per the government’s policy of devolution, Local Government Authorities (LGAs) are responsible for operating and managing primary-level health services, while regional authorities supervise LGAs and manage regional hospitals. MoHCDGEC shares regulatory and accountability functions with the President’s Office for Regional Administration and Local Government (PO-RALG) [16].

### Study design

This study adopted a descriptive case study approach well-suited to investigating a phenomenon in real-life settings [17]. A stratified sampling technique was used to select districts to include in this study. Tanzania includes eight health zones: Eastern, Central, Lake, Southern Highland, Southern, Northern, Southern West Highland and Western. In the first step, four health zones were purposively selected with consideration of variation in geographical representation. From each of these four zones, one district was randomly selected for in-depth analysis: Lushoto (Northern zone), Kilwa (Southern zone), Ikungi (Central zone) and Iringa (Southern Highland zone). Table 2 provides an overview of key demographic and health characteristics of the four study districts.

**Table 2:** Key demographic and health characteristics of the study districts

Key Indicator	Iringa	Ikungi	Lushoto	Kilwa
Population	254,032	272,959	332,436	200,015
Population growth rate	1.6%	2.4%	1.1%	0.9%
Hospitals	1	2	2	2
Health Centres	6	3	5	5
Dispensaries	61	32	46	47
Divisions	6	4	5	6
Wards	25	28	33	23
Villages	143	101	125	91
Health workers available^a^	37%	39.5%	43%	31%
Shortage of health workers^a^	63%	60.5%	57%	69%

Source: CCHPs (2017/2018); Census Report 2012; ^a^ Based on the Ministry of health human resources for health data 2014

### Data collection

To explore the design and implementation of SAs at the district level, we conducted in-depth interviews with a range of key informants and stakeholders. At national level, these included officials in the Ministry of Health, the PO-RALG, development partners and Christian Social Services Commission (CSSC), an umbrella organization that coordinates Christian faith-based health providers. At regional and district levels, key stakeholders included Regional Medical Officers, Council Health Services Board, District Medical Officers, Council officials, and management teams at faith-based health facilities. Purposive and snowball sampling techniques were used to identify interviewees. All respondents who were approached agreed to participate in the study. As indicated in Table 3, 39 interviews were carried out, including six interviews at national or regional level and 33 interviews at district level. We developed our own interview guide, informed by the topics that comprise the Walt and Gilson framework. Interviews were conducted in Kiswahili language by SM, DC and CM in 2016. All interviews were audio-recorded after obtaining verbal permission from the respondents.

**Table 3:** National, regional and district level key informants

	District level respondents	Number of interviews			
		Ikungi	Lushoto	Iringa	Kilwa
1	Council Health Management Team	3	3	4	3
2	The Council Health Services Board	1	–	1	1
3	Diocese Leaders (Bishops’ offices)	1	2	1	1
4	District Legal Officers	1	–	–	1
5	FBOs providers and administrators	2	2	2	2
6	Hospital financial officers	–	1	1	–
	Total District level interviewees	8	8	9	8
	National and regional level respondents	Role in the Service Agreement			
1	Ministry of Health, Community Development Gender, Elderly and Children (former Ministry of Health & Social Welfare)	-Formulates SA template (policy) and monitors the implementation of this policy -Finances service agreement			1
2	President’s Office Regional Administration and Local Government	Formulates SA template and monitors the implementation of this policy			1
3	Christian Social Services Commission (CSSC)- Umbrella Organization for Christian faith-based organisations	Provides technical support to health facilities under their umbrella that have entered into SA with the district councils			1
4	Development partners	Provide technical and financial support in the development and implementation of the SA			1
5	Regional Health Management Team	Provides technical back up to the district councils in the implementation of the SA			2
	Total interviewees				6

In addition to the interview data, we reviewed various documents, including guidelines for developing SAs, signed contracts, and hospital annual reports. The document reviews were primarily used to supplement and cross-check information on the nature and content of the contracts, including the types of services covered, how contracts were financed, contract management and performance monitoring mechanisms.

### Data management and analysis

The recorded interviews were transcribed verbatim by experienced transcribers and were checked for accuracy by four research team members (SM, DC, ED and CM). The interview transcripts were then translated from Kiswahili into English by a professional translator and the translations checked for accuracy by the Principal Investigator (SM). The first four authors (SM, DC, ED and CM) each read between five and 10 transcripts to familiarize themselves with the data. Two members of the research team (SM and DC) developed a code manual based on the objectives of the study and conceptual framework. The codebook was shared for review with senior researchers (ZS and KR). Using NVivo10 qualitative data analysis software [18], three members of the research team (SM, DC and CM) independently coded the first five interviews to develop consistency. Thereafter, SM and DC coded the remaining transcripts. New codes that emerged during the coding process were added with consensus from all research team members. Saturation was achieved when no more codes emerged from the data. Key themes were then independently identified by each of the coders and organized by level of respondent to facilitate comparisons. The themes were discussed by the researchers. Finally, two researchers (SM and DC) identified representative quotations for each key theme, and obtained consensus from all team members. The study thus used deductive and inductive methods to generate the themes [19]. The key dimensions from Walt & Gilson’s policy analysis framework—actors, process, context and content—informed the deductive approach to analysis and the format for reporting our findings in the following section.

## Results

### The policy process and actors involved

This section describes the policy process, including key actors involved in the design and implementation of the SA reform, at the national and district levels.

#### How did the policy emerge and evolve at the national level?

Document analysis and national level interviews alike indicated that engagement between the government and FBOs evolved over time and was broadly influenced by domestic and international socio-economic changes. In particular, the policy and institutional frameworks for SAs are rooted in the history of public-private sector collaboration in provision of health care services. Since Tanzanian independence, various semi-formal and informal arrangements have existed between the government and faith-based NSPs. However, until 1992, no formalized system defined partnerships between government and NSPs [20].

In the 1990s, the introduction of the health sector reforms agenda occurred as the government struggled to deal with economic crises that adversely affected health services delivery. During this period, the concept of partnerships between government and NSPs in health services delivery gained importance. The 1990 National Health Policy underlined the need for the active participation of NSPs in the provision of health services [21]. In 1992 the government formally negotiated agreements providing bed and staff grants to hospitals managed by FBOs [6]. The 1994 formulation of the health sector reforms (HSRs) policy represented the next major milestone. The HSRs highlighted the importance of, among other things, the role of NSPs in health service delivery [22]. In 2005, the government revised the 1992 agreements so that contracts could be signed at district level by the office of the District Executive Director (DED) rather than only by the Ministry of Health, as had been the case previously.

In 2007 the government introduced the Primary Health Services Development Program (PHSDP), a major initiative to run from 2007 through 2017 [23]. This program also recognized the role of NSPs in expanding the coverage of health services. Consequently, in the same year, the Ministry of Health and Social Welfare (MoHSW) (since renamed MoHCDGEC) developed a SA template to guide contractual arrangements between NSPs and the local government authorities [24]. The template was developed collaboratively by a steering team that was led by the MoHSW. Other stakeholders on the steering team included the Association of Private Health Facilities in Tanzania, CSSC, National Muslim Council of Tanzania, Tanzania Public Health Association, and DPs such as the Tanzania-German Programme to Support Health (TGPSH), the Danish International Development Agency (DANIDA) and the United States Agency for International Development (USAID) [14].

While the Ministry of Health led the process, DPs provided significant technical and financial support to formulating, drafting and refining the SA template, in addition to advocating for the reform. One DP respondent said:

*Apart from advocating for the public-private partnership and providing technical advice to the Ministry of Health, we participated actively in the drafting of the service agreement template at the national level.* (KI#36_Development partner)

Another respondent also commented on the inclusion of development partners:

*The Ministry of Health was in the forefront in formulating the service agreement template. However, we got high technical support from the development partners, particularly TGHS, DANIDA and USAID. They even participated in writing the policy.* (KI#34_Umbrella organization)

In 2009, Tanzania’s first public-private partnership (PPP) policy was developed to guide the institutionalization of these partnerships [24]. The PPP Act followed in June 2010 [25] and PPP Regulations were gazetted in June 2011 [26]. Other related policy documents include the MoHSW PPP strategic plan 2010–2015 [27] and the MoHSW PPP policy guideline [28].

#### How were the service agreements adopted at district level?

Per these policies, responsibility at the district level for developing and signing SA contracts was vested in the Council Health Management Team (CHMT) and the DED’s office, as well as the managers and owners of NSP health facilities [14].

The actual process of developing the SA typically entailed a number of activities. The district government formed a team of experts to carry out contract development. Several consultative meetings between local government officials and FBOs were conducted. The local government team consulted with the Ministry of Health and received technical support from development partners, particularly GIZ, which had established a presence in four regions in Tanzania. One development partner noted:

*We actively participated in advocating for the service agreement to the districts and non-state providers. We aimed to make district officials aware of the need for the service agreement and to strengthen their capacity in developing and implementing the contracts. Even looking at the number of signed service agreements to date, almost half are in four regions supported by us.* (KI#36_Development partner)

The FBOs, for their part, received technical support from the CSSC umbrella organization and the development partners.

### Contextual factors influencing the service agreements

The demand for contractual arrangements emerged from both the government and the FBOs. All contracted health facilities were located in areas where there were no publicly-owned hospitals. In these hospitals, patients had to pay for health services, including maternal and child health (MCH) services that were provided free of charge in publicly-owned health facilities. The majority of people could not afford these services and consequently travelled long distances to access public hospitals [14]. The government sought to increase access to affordable health care services for the population, particularly where government-owned health facilities were not available. The SA represented an opportunity for the government to provide financial support to existing NSPs, thereby meeting its goals of expanding access to affordable health care services to the general population and free services to selected populations. One district-level respondent described this situation:

*We did not have a public-owned hospital in our district. While this [faith-based] hospital existed, people could not afford paying for the services. They had to travel very far for the public health facilities. We saw it important to negotiate with our colleagues in order to increase access to services, particularly to women and children.* (KI#05_District health manager)

Another respondent had a similar comment:

*For example, people living near [the] Mission Hospital could not afford paying for the services. They had to travel to [the] district hospital*, *which is more than 20 km away. This not only increased costs but also contributed to high maternal and child deaths. It was important for the district to sign the contract with [the Mission] Hospital in order to increase access to services, and more importantly, provide free maternal and child health services.* (KI#18_District health manager)

Contracting-out also met needs of the FBOs. They were facing increased demand for health services and declining financial resources from donors. Interviews with district health managers and the FBOs alike revealed that in the early 2000s most FBOs were encountering challenges in providing health services. With declining donor support, the FBOs could not generate enough resources to meet the demand for medicines, equipment, infrastructure maintenance and salaries of health care personnel. The government, meanwhile, had improved the salaries and incentives provided to personnel in the public health sector. As a result, health professionals, particularly medical doctors and nurses, were leaving faith-based hospitals to join public hospitals. Faith-based hospitals, therefore, needed new sources of financial and human resources in order to effectively provide health services. One hospital administrator noted:

*I was one of the management team members and it was a time when there was big exodus of health workers. Doctors were moving away to the government hospitals and we found that we needed some help from the government. Therefore, we decided to negotiate with the district council to see how it could help us, and by then the district had no district hospital.* (KI#06_In-charge FBO facility)

### The content of the service agreement policy

This section describes the nature and content of the SA policy, including the type of services the contracts covered, target populations, financing, and accountability and performance monitoring mechanisms.

#### Types of services covered in the contracts

In all districts, the contractual arrangements mainly aimed at increasing access to MCH services. The central government had committed to provide free MCH services in all public health facilities. The specific services covered included antenatal care, delivery and postnatal care services and prevention of mother-to-child transmission of HIV [14]. The districts were, therefore, obliged to ensure that patients receiving these MCH services were not charged.

In addition to free MCH services, contracted hospitals were required to subsidize health services for the general population. The contracts required FBOs to follow the government’s price list from the Cost Sharing Guideline of 1997. However, hospitals did not consistently adhere to the recommended health service prices. Our review compared the Cost Sharing Guideline with actual hospital price lists; findings from these document reviews were confirmed in interviews with officials from local government and contracted FBOs. Many hospitals set their own prices higher than the prices in the Cost Sharing Guideline. In interviews, officials attributed this inconsistency to insufficient monitoring of the SAs.

#### Financing of the service agreements

In two of the four districts where this study was conducted, the contracts were initially funded by GIZ, which disbursed funds to district authorities. The contracted hospitals were paid on a fee-for-service basis by the district authorities for services rendered to pregnant women, children and other vulnerable groups of the population. One respondent described:

*Initially, we received forty million [Tanzanian] shillings from the Tanzania Germany Programme for Health Support (TGPHS) to finance the service agreement. They promised that if funds were used efficiently, they would fund the contract for another year.* (KI #26_FBO provider)

However, GIZ’s financial support ended when its grant closed out and the districts were not able to finance the contracts with locally-generated resources. Both districts then changed the mode of payment from fee-for-service to a lump sum. A similar lump-sum financing mechanism was reported in the two districts that were never supported by donors. Districts were required to include a budget for contracted hospitals in the district annual health plans, commonly known as Comprehensive Council Health Plans (CCHPs). These were financed by the central government using the HBF. The basket fund, while administered by the central government, is itself largely dependent on donor support [15]. HBF funds were allocated according to a formula determined by the central government: population size (70%), poverty count (10%), district medical vehicle route (10%), and preventing under-5 mortality (10%) [14]. Funds were disbursed to contracted NSPs from district councils on a quarterly basis. The SAs required districts to allocate 25% to 30% of their annual health plan budgets to the contracted hospitals and contracted NSPs were supposed to be actively involved in the planning and budgeting process. However, the contracted FBOs felt that they were inadequately involved during planning and budgeting. Respondents reported that they were only involved during the preparation of CCHPs for the basket fund. Other activities implemented by contracted FBOs but using sources other than the basket fund were not jointly discussed. In addition to the HBF, contracted FBOs received other support including staff training grants from the central government, seconded staff from district councils and contributions of medicines and medical supplies from the Medical Stores Department (MSD) of the Ministry of Health, as reported by one respondent:

*Since we signed the service agreement with the district, we have been getting support from the central government through the district. Some staff in our hospital are paid their salary by the central government. We also receive staff from the district and funds for medicines through the MSD.* (KI#04_Dioces leader)

Another respondent added:

*We are getting support from the government in terms of staff, salaries, as well as allocation of medicines and medical supplies through the medical stores department.* (KI#24_ FBO health provider)

Two main problems were reported by the FBOs in relation to financing: overall shortages of funds and delays in the disbursement of funds. Shortages were caused by high demand for health services. Contracted FBOs regularly reported that they had served more MCH clients than before SA was in place, including some clients coming from neighboring areas. This increased staff workload and incurred extra costs for these health facilities, as reported by staff at the FBOs:

*Patients who come for health services in this hospital do not only come from [this] district. Other patients come from neighboring districts. We are working at a loss because we are spending more than we are receiving.* (KI#8_FBO provider)

Another respondent stated:

*The support we get from the government is not adequate. We receive patients even from outside the catchment area. But the contract says that you get budgetary allocation in the basket fund according to the catchment area which you serve. So a challenge emerges in the sense that the service which you provide and that which is covered become an issue.* (KI# 10_ FBO provider)

However, district health managers argued that the government support was meant to complement, not replace, other sources of hospital revenues. District health managers raised concerns that FBOs were not transparent about their other sources of income, such as user fees, cost sharing, insurance and receipts in-kind in their respective health plans. One DHM said:

*The hospital needs to be transparent. Our fellows [i.e. the contracted hospitals] do not disclose the incomes generated from other sources. They only report expenditures related to basket fund. It would be good if they also disclosed the income generated from other sources.* (KI#09_District health manager)

Another government respondent felt similarly:

*The main problem with our partners is transparency; transparency in matters of the income which they get, for example, from their donors. You will find that they read the collections statement to the Board, but transparency in resources which they get from other sources is not there.* (KI#32_Regional health manager)

Disbursing funds from the contracts also created challenges. The government was supposed to disburse funds to hospitals quarterly. However, significant delays were reported by many:

*The money from the Government usually comes late. It may be the case that it is a problem from the top, but when it comes we already have used our own resources, and we get stuck in one way or another.* (KI#06_In-charge of FBO hospital)

Another respondent noted:

*Delays in the disbursement of funds is a serious problem. Although the funds provided are not adequate, if they were disbursed on time they would help overcome financial crisis in the contracted hospitals.* (KI#36_Development partner)

In their interviews, district officials reported that the delays in disbursing funds to the hospitals were due to delays in receipt of funds from the central government. Ministry of Health officials likewise reported that delays in disbursing basket funds to the districts were caused by delays in receiving funds from donors.

*We largely depend on funds from the development partners. The delay is sometimes due to late receipt of funds from the development partners. This is a big challenge for our country* (KI#37_National level respondent).

#### Management of contracts

The MoHCDGEC is primarily responsible for the formulation of the contracting-out policy, advocacy for the initiative and monitoring implementation. At the national level, a public-private partnership (PPP) office (desk) at MoHCDGEC headquarters coordinates PPP arrangements in the health sector. Another PPP desk at the PO-RALG headquarters coordinates PPP matters in all sectors. Both PPP desks liaise with regional PPP forums and steering committees. These, in turn, are supposed to provide technical back-stopping to district councils in the implementation of the SA, including linking the MoHCDGEC with the district councils for reporting [14, 28].

The SAs clearly stipulate conditions, duties and obligations for local government authorities and NSPs alike. According to the SAs, contracted FBOs are eligible to receive funds only following submission of quarterly technical, financial and progress reports. The Regional Health Management Team and the CHMT are responsible for overseeing technical implementation of the SA. The CHMT members have the authority to conduct spot checks in contracted facilities. In all districts, day-to-day management of the SA contract was part of the mandate of hospital boards composed of members from government and the FBOs. A hospital board, created for the purpose of SA management, is supposed to convene quarterly [14, 28].

However, interviews revealed inadequate capacity of the contracted parties to implement the contracts. This perspective was elaborated by respondents from multiple sectors, beginning with development partners:

*The main challenge I see is the capacity of both parties to manage the contract. On the one hand, most of the contracted hospitals have inadequate capacity in terms of human resources. Very few staff are competent and able to oversee the implementation of the contract. On the other hand, the district health managers are not capable to oversee the delivery of quality health services in the contracted hospitals.* (KI#36_Development partner)

A FBO respondent reported a similar experience:

*The district health managers were supposed to conduct supervision on quarterly basis and submit reports to the hospital board. Unfortunately, supervision is rarely conducted and board meetings are not held regularly. This makes it difficult to detect and address challenges in the implementation of the service agreement.* (KI#28_Diocese leader)

According to the guidance in the contracts in all districts where this study was conducted, contracts were supposed to be reviewed after every three to 5 years. This study was conducted nearly 10 years after the contracts were established, but no district had reviewed the contract.

The SAs provided no guidance on dealing with disagreements. Conflicts among the parties were expected to be solved amicably, and little recourse was available when they could not be resolved. Inadequate accountability mechanisms made it difficult for both the local government and the NSPs to take action when intractable conflicts arose.

*There is big control of us. If the government does not provide the money that we are spending for maternal and child health*, *we have no mechanism to make the government accountable for it. We need to have mechanisms for making the government accountable for it.* (KI#04, In-charge of FBO hospital)

## Discussion

This study explores the contracting strategy used to engage NSPs in the Tanzanian government’s efforts to move towards universal health coverage. Most of the existing literature on contracting-out focuses on assessing impact, rarely describing specific design and implementation features in detail. The study adds new knowledge on the processes by which NSPs were engaged in the context of a resource-poor setting. The context in which contracting-out is implemented and the design features of the intervention greatly influence its chances of success [10, 11]. The lessons learned in this study regarding contracting policy design and implementation could be relevant to future efforts in Tanzania as well as to other countries implementing contractual agreements between governments and NSPs to improve primary health care services. The paper also provides some reflections on the use of the Walt and Gilson framework.

### Building contracting-out into existing policy and practice

Previous involvement of NSPs in the national health system was mainly founded on mutual knowledge and personal, trust-based relationships. The introduction of service agreements as a mechanism through which the Tanzanian government engaged FBOs in providing primary health care services added formality to the process of contracting out. Official Acts, policies and guidelines [25–28] were established that institutionalized and standardized what the agreements covered and how services operated. These legal and policy frameworks and structures facilitated effective contracts to assure the provision of primary health care services according to government standards.

While the formality of the new service agreement offered more guidance and guaranteed more accountability, relationships and trust among different actors at the national and district levels remained influential. These extended beyond the parties named in the SAs. For instance, trusting relationships existed or were built among: key stakeholders in the Ministry of Health who were responsible for policy guidelines and quality assurance; staff at PO-RALG who were responsible for policies at the district level; development partners providing financial and technical support; and the government stakeholders leading the process. Likewise, existing trust between the public and the NSPs in districts were central to encouraging people to seek health services at the institutions.

In Tanzania (as in many other developing countries), development partners actively influence policy design and implementation processes [29]. The study’s findings revealed that international partners have played a significant role in placing contracting-out on the HSR agenda, as well as in the SA policy design and implementation. This has long-term implications. The government of Tanzania remains heavily dependent on donor funding for health care expenditures, including financing for SAs. Studies elsewhere have indicated that while the support provided by DPs is significant and highly appreciated, it can create problems related to sustainability of the relevant policies and interventions [30–32].

Further, dependence on donor financing and technical support leaves domestic policy processes open to external influence. This can result in a negotiated set of priorities that reflect technical, political, and economic considerations defined more by the interests of donors than domestic needs [33, 34]. Concerns about the impact of donor dependence can be alleviated when the central government builds sufficient internal technical and financial capacity to meaningfully participate in negotiations and to support district authorities as they establish and finance contractual agreements with NSPs.

### Implementing contracting-out policy

With policy guidance and technical capacity in place, implementation becomes the next challenge. Our study revealed that district leaders did hold real authority when negotiating contractual agreements with NSPs. This was a significant difference from earlier models of contracting-out in Tanzania reported in other studies. In these earlier models, contractual agreements were made centrally by the Ministry of Health and district-level authorities were left out of the choice of NSPs and contract negotiation processes [6].

Financial management remained a problem with SAs. While the districts now had both the mandate and the power to make contractual agreements with the NSPs, they still had little power over the financing of the contracts, nor could they finance them directly with their own resources. Districts depended on the central government to provide financing for the SAs through basket funding from donors. Insufficient and untimely payments negatively affected the implementation of the contractual agreements. We found wide agreement among our respondents that contracted FBOs were compelled to compensate for financing gaps through their own or other external resources; these continued to become increasingly limited. FBOs in Tanzania reported facing growing difficulties resulting from decreased external financial support. Financial management difficulties and gaps had serious negative effects for the faith-based NSPs. This finding corroborates assessments of contracting-out experiences in Cameroon and Chad, as well as other experiences from Tanzania [6]. For example, in Cameroon it was reported that the Ministry of Health did not fulfil commitments on subsidies, allocation of staff, and official recognition of hospitals as district hospitals, despite repeated requests from NSPs [6]. Likewise, a recent study in other districts in Tanzania reported significant shortages and delays in disbursements of funds from the central government to NSPs [35]. Recent studies on decentralization in Tanzania have indicated that transferring decision-making powers without fiscal power can lead to sub-optimal outcomes [36, 37].

The inclusion of the non-state sector in budgeting and planning processes at all levels is fundamental for strong PPP relationships. Participation by NSPs leads to more efficient and effective use of available resources, especially in district-level annual health plans. However, the private sector allocation in CCHPs remained a constant 25%, without accounting for variations in available providers or level of need in a given district. More comprehensive planning and mapping of resources throughout the sector—both thematically and geographically—could facilitate improved equity in resource distribution. Moreover, the limited capacity of district governments to make timely payments to contracted NSPs may drive deterioration of the relationships between the government and NSPs [12]. The government and the contracted NSPs must maintain continuous dialogue to ensure clear expectations of roles and responsibilities. Ongoing dialogue would also allow the parties to quickly address and resolve any misunderstandings that occur during the implementation of the SAs.

Implementation of SAs (and other PPP arrangements) required skill sets that were not necessarily available among either district councils or the NSPs. Other assessments of the SAs in Tanzania have reported that needs assessments were not conducted prior to signing agreements; they also found that monitoring and evaluation were not adequately done by the government [35, 38]. A comparative study on contractual agreements between the government and faith-based health providers in Cameroon, Chad, Uganda and Tanzania reported similar challenges and their negative effects on the contracting experiences in these countries [6]. Another study, conducted in Malawi, concluded that Service Level Agreements (SLAs) between the government and the FBOs were introduced too quickly, before adequate supporting structures, such as clear policies to guide implementation or arbitration committees to resolve difficulties, had been established [39]. This resulted in growing mistrust, moral hazard, and in some cases the termination of SLAs. Building public sector capacity to work with the private sector, including developing skills to negotiate and oversee contracts with private providers, is imperative.

The success of contracts often depends on whether they create a sense of accountability in addition to formal requirements for monitoring adherence and providing information to improve services as needed. This study indicated that the lack of mechanisms for monitoring resulted in hospitals setting prices for services which exceeded those in the contract. The NSPs argued that the absence of a review mechanism for the SAs forced them to increase prices to reflect increasing costs and the changing economic context. This highlights the risks of implementing contracts for long periods without review. Studies in other settings also reported on several contracting projects that suffered as a result of poor monitoring [40–43]. These findings suggest that the central government must play a role beyond overall strategic policy leadership and financing of health care. All levels of government should be required to monitor health care delivery in order to remain up-to-date with the situations faced by providers.

### Using the policy triangle analysis framework

The Walt and Gilson policy analysis framework helped organize and simplify our study of a complex set of key factors (actors, processes, content and context) and their interrelationships in policy creation. The use of this framework particularly guided the study’s approach to analyzing the socio-economic, political and international contextual factors and actors that influenced the process by which the SA policy was designed and implemented. The framework also made it possible to analyze how the content of the SA policy fulfilled its objectives [44–46]. The policy triangle framework is recommended to researchers seeking to understand complex policymaking and implementation processes [44, 46]. Knowledge generated from this policy analysis may be useful to researchers and other stakeholders seeking to influence policy-making in LMICs [12, 44]. Further, using the same framework to study multiple settings enables future cross-country or time-series analyses.

### Limitations of the study

This study relied primarily on document reviews and interviews with stakeholders involved in the development and implementation of the service agreements at the district level. The study did not, however, interview any of the intended beneficiaries of the SAs to assess their experiences and perceptions of the health services provided. Secondly, the study was limited to four districts due to budget and time constraints. While efforts were made to sample districts with varying characteristics and respondents involved at different levels of decision-making, the results may not be generalizable to other districts or contexts.

## Conclusion

Strengthening PPPs in primary health care is essential to achieving universal health coverage in Tanzania. Introducing service agreements as a mechanism for contracting-out public primary health care services in Tanzania successfully gave districts the mandate and power to make contractual agreements with NSPs. However, financing the contracts remained largely dependent on donor funds via central government budget support. The limited financial control held by the districts undermined effective implementation of the SAs with faith-based health NSPs. NSPs must be more fully involved in district annual health plans and in health budgeting and planning processes at all levels. Meaningful involvement of NSPs should lead to more efficient and effective use of limited available resources. Further, the central government needs to continue building its own and district-level capacity to provide technical and financial support to districts establishing contractual agreements with NSPs. Finally, continuous dialogue is needed between the various parties, including government, donors and contracted NSPs. Communication and dialogue reinforce the trust-based relationships that ensure clear expectations for each party and enable the parties to resolve misunderstandings or other disagreements that arise during the implementation of service agreements. Tanzania’s implementation of SAs in contracting-out delivery of primary health care services has already made significant contributions towards the country’s movement for universal health coverage. Lessons learned during the processes of the SA policy development and implementation can be applied to further strengthen and streamline partnerships among state and non-state actors for health.

## Abbreviations

CCHP: Comprehensive Council Health Plan
CHMT: Council Health Management Team
CSSC: Christian Social Services Commission
DDH: Designated District Hospital
DED: District Executive Director
FBO: Faith-Based Organization
GBS: General Budget Support
GIZ: Deutsche Gesellschaft für Internationale Zusammenarbeit
HBF: Health Basket Fund
KI: Key Informant
LGA: Local government authority
LMICs: Low and Middle Income Countries
MCH: Maternal and Child Health
MoHCDGEC: Ministry of Health, Community Development, Gender, Elderly and Children
MoHSW: Ministry of Health and Social Welfare
MSD: Medical Stores Department
NGO: Non-governmental Organization
PHSDP: Primary Health Services Development Programme
PMTCT: Prevention of Mother to Child Transmission of HIV
PO-RALG: President’s Office - Regional Administration and Local Government
PPP: Public Private Partnership
RMO: Regional Medical Officer
SA: Service Agreement
SLA: Service Level Agreement
TGPSH: Tanzania German Programme to Support Health
UHC: Universal Health Coverage
WHO: World Health Organization
